# Long-term comparison of maxillary protraction with hybrid hyrax-facemask vs. hybrid hyrax-mentoplate protocols using Alt-RAMEC: a 5-year randomized controlled trial

**DOI:** 10.1093/ejo/cjaf011

**Published:** 2025-03-19

**Authors:** Joeri Meyns, Jeroen Meewis, Flore Dons, Arnoud Schreurs, Johan Aerts, Sohaib Shujaat, Constaninus Politis, Reinhilde Jacobs

**Affiliations:** Department of Oral and Maxillofacial surgery, General Hospital St-Jan, Synaps park 1, 3600 Genk, Belgium; Department of Imaging and Pathology, OMFS-IMPATH Research Group, Faculty of Medicine KU Leuven, Kapucijnenvoer 33, 3000 Leuven, Belgium; Department of Oral and Maxillofacial surgery, General Hospital St-Jan, Synaps park 1, 3600 Genk, Belgium; Department of Oral and Maxillofacial surgery, General Hospital St-Jan, Synaps park 1, 3600 Genk, Belgium; Private Practice, Weg naar As 172/1, 3600 Genk, Belgium; Private Practice, Collegelaan 12, 3600 Genk, Belgium; Department of Imaging and Pathology, OMFS-IMPATH Research Group, Faculty of Medicine KU Leuven, Kapucijnenvoer 33, 3000 Leuven, Belgium; King Abdullah International Medical Research Center, Department of Maxillofacial Surgery and Diagnostic Sciences, College of Dentistry, King Saud bin Abdulaziz University for Health Sciences, Ministry of National Guard Health Affairs, Ar Rimayah, Riyadh 11481,Kingdom of Saudi Arabia; Department of Imaging and Pathology, OMFS-IMPATH Research Group, Faculty of Medicine KU Leuven, Kapucijnenvoer 33, 3000 Leuven, Belgium; Department of Imaging and Pathology, OMFS-IMPATH Research Group, Faculty of Medicine KU Leuven, Kapucijnenvoer 33, 3000 Leuven, Belgium; Department of Dental Medicine, Oral Facial Diagnostics and Surgery,Karolinska Institute, Alfred Nobels Allé 8, Huddinge, Stockholm, 141 50, Sweden

**Keywords:** skeletal class III malocclusion, maxillofacial protraction, orthodontics, orthopedic therapy, bone anchored, facemask

## Abstract

**Background:**

The study aimed to compare the short- and long-term effectiveness of hybrid Hyrax (HH) -Facemask (FM) and HH-mentoplate (MP) treatment protocols for maxillary protraction using Alt-RAMEC.

**Methods:**

A single-center 2-arm parallel randomized controlled trial. *Participants:* 28 skeletal class III patients (female: 14, male: 14; average age: 9.7 ± 1.3 years;) were included. *Interventions:* Two treatment groups where protraction therapy was combined with Alt-RAMEC. Group 1: Facemask group (Hybrid Hyrax + Facemask) and Group 2: Mentoplate group (Hybrid Hyrax + Mentoplate). *Objective: To compare skeletal and dental changes between groups using low dose computed tomography (CT) scan from which virtual lateral cephalograms were generated. Outcome:* Outcomes include changes in Wits appraisal (primary outcome), and cephalometric analysis of skeletal and dental changes (secondary outcomes) at 1 year and 5 years after treatment initiation. *Randomization:* 28 patients were allocated to either treatment-protocols using sequentially numbered opaque, sealed envelopes. The randomization sequence was generated with a 1:1 allocation ratio. *Blinding:* Due to the nature of the trial, the operator and children could not be blinded to the treatment allocation. However, blinding was used when assessing the outcomes.

**Results:**

*Follow-up:* one patient was lost at the one-year follow-up and an additional three patients were lost at the 5-year follow-up. *Outcomes:* Both treatment protocols effectively improved intermaxillary relationship. Wits measurements showed improvements of 4.42 mm (FM) and 2.86 mm (MP) at T1, decreasing slightly to 3.33 mm (FM) and 1.50 mm (MP) at T2. While vertical control and incisor inclination were comparable between groups long-term, short-term differences were noted in upper and lower incisor inclination. Results remained equally stable after five years (T2). *Harms:* minor harms were encountered with the anchor hooks (fracture or mucosal irritation), however none led to treatment cessation

**Conclusions:**

Early class III treatment with HH + MP provided similar outcomes and stability to that of HH + FM suggesting that the choice between FM and MP should be based on individual patient factors rather than presumed mechanical advantages.

**Trial registration:**

Clinical Trials ID: NCT02711111

## Introduction

### Background

The treatment of growing skeletal Class III patients is often viewed as one of the most challenging orthodontic issues. This is due to the unpredictable growth potential of the maxilla and the possibility of unfavorable mandibular growth. It is difficult for an orthodontist to predict the magnitude and timing of skeletal growth. Moreover, the eligibility of patients for early Class III treatment remains a subject of debate [1–3]. One of the most prevalent orthopedic treatment options for early Class III correction is facemask (FM) therapy alone or in combination with rapid palatal expansion (RPE) appliance. However, this method has undesirable side effects such as dentoalveolar compensation, minimal skeletal effect, and unwanted vertical changes [4].

In recent years, several techniques for orthopedic treatment with skeletal anchorage devices have gained popularity [5–18]. These devices have been suggested to reduce the adverse effects associated with FM therapy and even successfully treat large Class III deformities [3, 5, 6, 10, 19–26]. Despite the widespread use of skeletal anchorage for interceptive Class III treatment, there is currently no consensus on indications, techniques, age, protocols, or forces employed [2, 27, 28]. Furthermore, skeletal anchorage procedures do come with possible drawbacks: they involve more or less invasive procedures to place and subsequently remove the devices. Also, some miniscrews and bone anchors are not stable throughout treatment [10, 29]. Whether bone-anchors provide better long-term stability of the treatment effect is unknown, due to the lack of evidence at this moment [27, 30, 31]

Amongst the anchorage devices, Hybrid Hyrax appliance (HH) has been successfully implemented, which uses two mini-implants in the anterior palate to provide skeletal anchorage for maxillary protraction during simultaneous rapid palatal expansion (RPE) [32, 33].

The effectiveness of Rapid Palatal Expansion (RPE) for maxillary protraction remains debated [34, 35], though it may enhance skeletal effects by mobilizing midfacial sutures. The Alternate Rapid Maxillary Expansion and Constriction (Alt-RAMEC) protocol claims to achieve greater and faster skeletal changes by further stimulating upper jaw growth through circummaxillary suture opening [36, 37]. Meta-analysis shows that Alt-RAMEC combined with bone-anchored appliances produces superior sagittal skeletal effects while minimizing vertical and dentoalveolar changes [38]. However, recent research indicates that while skeletal anchorage in HH improves both sagittal and vertical skeletal effects, the addition of Alt-RAMEC to these devices shows no added benefit [33]. Therefore, the value of combining Alt-RAMEC with HH remains uncertain.

When considering the lower jaw, the use of bone-anchors, such as mentoplate (MP) in combination with maxillary HH [39–41] have also been hypothesized to produce more skeletal effect due to the direct transfer of force on the bone and potentially better patient compliance, however no evidence was found to support this theorem [3, 27, 30, 42]. Such a skeletal anchorage with symphyseal plates in the lower jaw could provide greater vertical control and might be the treatment of choice in high-angle patients [43–45]. However, lack of evidence exists assessing the efficacy of HH + MP in comparison to the conventional HH + FM therapy. Most studies on skeletal anchorage techniques are either clinical cases or case series, with a notable lack of randomized controlled trials (RCTs) [3, 27, 30, 31]. Furthermore, the long-term stability of the treatment effect provided by bone-anchors is yet to be explored [46].

### Objectives

Therefore, the objective of this prospective RCT was to compare the short- (1 year) and long-term (5 years) effectiveness of HH + FM and HH + MP therapy with Alt-RAMEC-protocol in growing Class III patients by assessing the CT-derived two-dimensional (2D) cephalometric variables.

## Methods

### Trial design

Single-center 2-arm parallel randomized controlled trial with 1:1 allocation ratio.

### Participants

This study examined patients with Class III skeletal malocclusion that were referred to our hospital by their orthodontist. All participants were in mixed dentition with anterior crossbite or an end-to-end incisor relationship and Class III molar relationship at start of treatment. Patients were excluded if they had cleft/craniofacial syndromes, prior orthodontic/surgical treatment, significant skeletal asymmetry, or functional Class III malocclusion. A single surgeon performed HH screw and MP placements, while three experienced orthodontists provided orthodontic care.

### Interventions

a. Palatal expansion by HH

**Figure 1.**
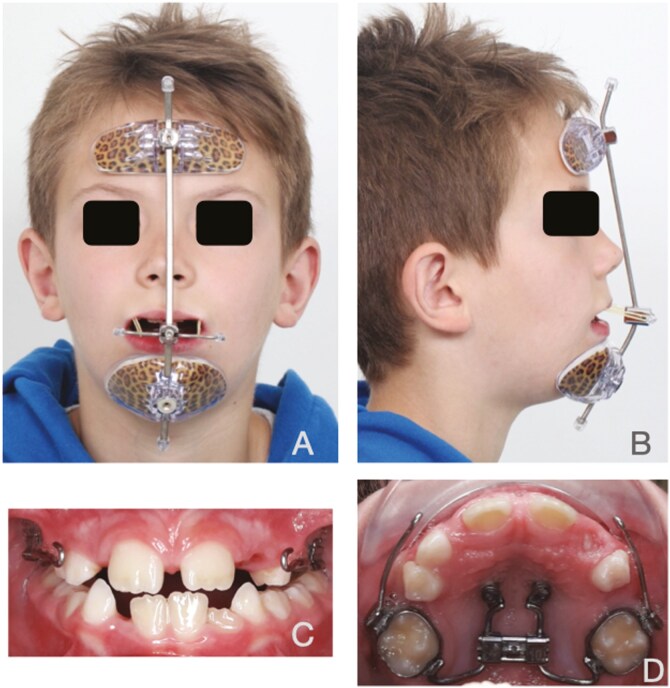
Facemask treatment. A. Frontal view; B. Lateral view; C. Intra-oral frontal view; D. Occlusal view hybrid hyrax.

**Figure 2.**
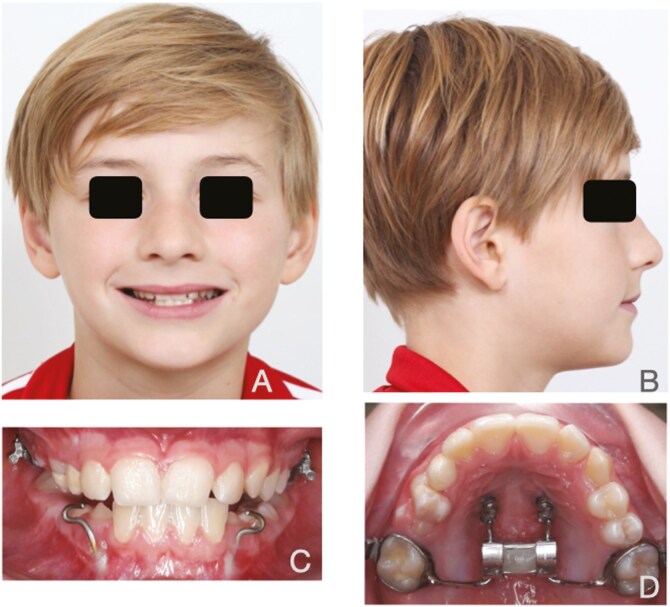
Mentoplate treatment. A. Frontal view; B. Lateral view; C. Intra-oral frontal view; D. Occlusal view hybrid hyrax.

In each patient, two self-drilling mini-screws (each 2 mm in diameter and 9 mm in length, sourced from Benefit miniscrews, PSM-medical solutions^®^, Gunningen, Germany) were implanted into the anterior palate around the third rugae. A Hybrid Hyrax (HH) apparatus was assembled with an expansion screw (Forestadent^®^) and affixed to bands. These bands were subsequently cemented to the first upper molars using a light-cured cement (Band-Lok, Reliance Orthodontic Products ^®^, Thorndale, USA) (Figs 1 and 2). The Alternate Rapid Maxillary Expansion and Constriction (Alt-RAMEC) protocol was employed [47], wherein the HH was activated by the patient’s parents twice daily (0.25 mm per turn, two turns in the morning and two turns at night) for one week, followed by deactivation twice daily (two turns in the morning and two turns at night) for the next week. This cycle of alternating activation and deactivation was repeated three times. In the following week, the maxilla was adjusted to the suitable transverse dimension.

b. FM group

In this group, Facemask (FM) therapy commenced concurrently with Alt-RAMEC. Elastics were connected from the hooks of the expander to the FM (Orthocomfort & Medical Distributors SL^®^, Barcelona, Spain), creating a downward and forward vector (Fig. 1). This configuration produced orthopedic forces of 360–400 g per side (equivalent to 12,7 – 14 oz). The patients were instructed to use the device for 12–14 hours daily, primarily overnight, for the initial six months or until they achieved a positive overjet of at least 2 mm. For the following six months, the FM was to be worn only during sleep. (Fig. 1)

c. MP group

Following general anesthesia, a mentoplate (MP) (PSM-medical solutions^®^, Gunningen, Germany) was inserted through a marginal gingival incision. The plate was bent and modified prior to fixation with two to four screws (KLS Martin^®^, Tuttlingen, Germany) (Fig. 2). The patients were simultaneously provided with Alt-RAMEC and protraction elastics, generating orthopedic forces of 185 g per side (equivalent to 6 ½ oz). The patients were advised to wear it continuously, 24 hours a day, 7 days a week, including during meals. They were also instructed to replace the elastics daily for the first six months or until a positive overjet of at least 2 mm was achieved. For the subsequent six months, the elastics were to be worn only during sleep. (Fig. 2)

d. Fixed appliances

During phase 1, only protraction therapy was used, with no fixed appliances present at T1. All patients later received identical full fixed orthodontic appliances (edgewise mechanics, MBT.022 slot, Empower R, American Orthodontics^®)^ during phase 2. Also no elastic traction was employed during phase 2, after the one year (T1) time-point. The treating orthodontists followed standardized techniques for both treatment phases.

### Outcomes

Outcomes include changes in Wits appraisal (primary outcome), and cephalometric analysis of skeletal and dental changes (secondary outcome) at 1 year and 5 years after treatment initiation. No changes were made after trial commencement.

a. Radiographic data acquisition

A single radiology technician performed low-dose CT scans at three intervals: baseline (T0), one-year post-treatment (T1), and five years post-treatment (T2). During scanning, patients lay supine with a wax bite in centric relation (in first tooth contact) and were instructed to remain still, breathe normally, and avoid swallowing. Scans were performed using a Somatom Force dual-source dual-energy CT system (Siemens^®^, Erlangen, Germany) with the following parameters: −0.6 mm slice thickness—0.3 mm increment—1.0 pitch—200 × 200 mm field of view—150 kVp tube voltage. The machine and parameter settings allowed better image quality and faster acquisition times, resulting in fewer motion artifacts and low radiation doses considering an automatic dose modulation protocol. Effective radiation doses for the applied CT were comparable to CBCT doses and ranged from 0.095 to 0.257 mSv per scan.

b. Cephalometric analysis

**Figure 3.**
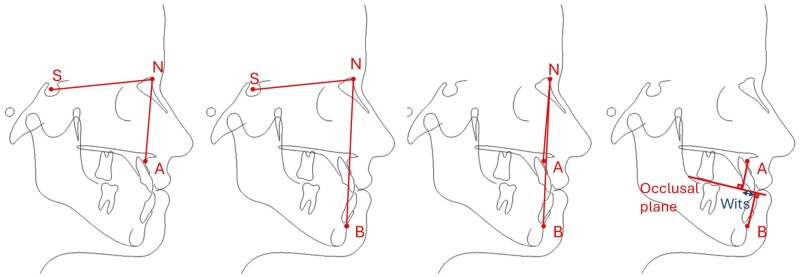
Sagittal cephalometric measurements evaluated in this study: SNA, SNB, ANB and Wits.

**Figure 4.**
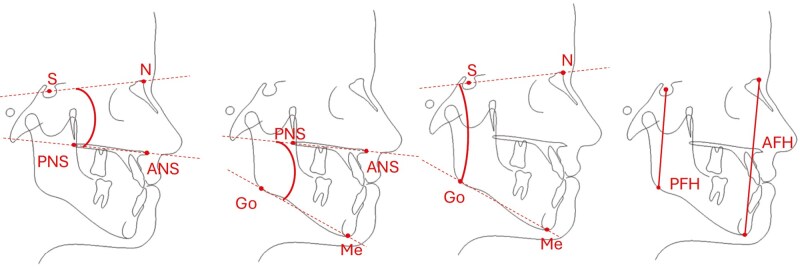
Rotational measurements evaluated in this study: SN-NL, ML-NL, SN-ML and PFH/AFH.

**Figure 5.**
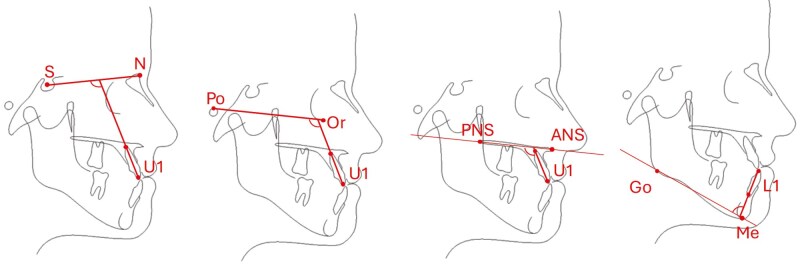
Dental measurement evaluated in this study: U1–SN; U1-FH; U1-NL; IMPA.

The cephalometric images were generated from the T0, T1 and T2 CT datasets with Planmeca Romexis software (version 6.3.0, Planmeca^®^, Helsinki, Finland), using an orthogonal method without magnification. The cephalograms were superimposed on the cranial base and cephalometric analysis was conducted at all time-points with the OnyxCeph software (version 3.6, Image Instruments GmbH^®^, Chemnitz, Germany). The cephalometric variables used in this study are listed in Table 2 and depicted in Figs 3–5. Two independent oral and maxillofacial surgery trainees analyzed 10% of the data twice at an interval of two weeks to determine the inter- and intra-observer reliability.

**Table 2.** Group characteristics at baseline (T0).

	Facemask Mean ± SD	Mentoplate Mean ± SD	^a^p-value
Sagittal			
SNA °	79.41 ± 2.43	78.38 ± 3.50	.38
SNB °	80.22 ± 2.14	78.94 ± 4.25	.33
ANB °	−0.82 ± 2.03	−0.55 ± 1.45	.69
Wits (mm)	−5.71 ± 1.82	−5.62 ± 2.36	.90
Vertical			
SN-ML °	31.71 ± 4.31	35.05 ± 6.08	.11
SN-NL°	6.49 ± 3.57	8.72 ± 4.32	.16
NL-ML°	25.2 ± 4.61	26.32 ± 5.84	.59
PFH/AFH	65.71 ± 3.89	63.00 ± 4.14	.09
Dental			
U1 -SN	105.53 ± 6.27	104.06 ± 9.27	.63
U1-FH	118.42 ± 6,70	115.70 ± 9.08	.38
U1 -NL	113.01 ± 5.65	114.47 ± 7.65	.58
IMPA	90.41 ± 5.21	91.40 ± 7.27	.69

^a^Intergroup comparison is performed using the unpaired two-sided samples t-test. Baseline comparisons are provided for descriptive purposes only, to confirm group equivalence following randomization, and should not be interpreted as inferential statistical analyses.

### Sample size calculation

When this trial began, no comparative studies of these specific techniques existed.

In 2010 Cevidanes et al. [5] conducted a controlled clinical trial comparing Bone Anchored Maxillary Protraction (BAMP) with Facemask and Rapid Maxillary Expansion (FM-RME), finding a mean Wits difference of 2.3 mm between groups. We pooled the standard deviations from both groups by first converting them to variances by squaring the standard deviations, then taking the average, and converting the average back to a standard deviation by taking the square root. A sample size calculation was performed for a one-sided t-test with a significance level of 0.05 and a power of 80%. This resulted in a required sample size of 12 patients per group. R version 4.1.2 was used, with the TrialSize library to calculate the sample size. We slightly overrecruited to account for potential dropouts.

### Randomization

a. Sequence generation

The randomization sequence was generated with a 1:1 allocation ratio. (for complete data, supplementary file 1)

b. Allocation concealment

Sequentially numbered sealed, opaque envelopes.

c. Implementation

The envelopes containing the allocation sequence codes were given to the patient by an intermediary and opened sequentially at the time of enrollment, excluding the clinician from the process.

### Blinding

Due to the nature of the trial, the operator and children could not be blinded to the treatment allocation. However, blinding was used when assessing the outcomes. This was achieved by pseudonymizing all patient data before and after treatment. The statistician analyzing the results was unaware of the group assignments.

### Statistical analysis

R version 4.1.2 was used, with the TrialSize library for statistical analysis. A normal quantile plot of the residual values showed that they were normally distributed. Homoscedasticity was tested visually by a residual dot plot. The inter- and intra-observer reliability of cephalometric analysis was evaluated using the Intra-Class Correlation Coefficient (ICC) at a 95% confidence interval. The data was descriptively analyzed using the median and standard deviation.

Unpaired two-sided samples t-test was employed for comparisons between two independent samples. A p-value of < .05 was considered as statistically significant. (Online repository for complete statistical code)

## Results

### Participants flow

**Figure 6.**
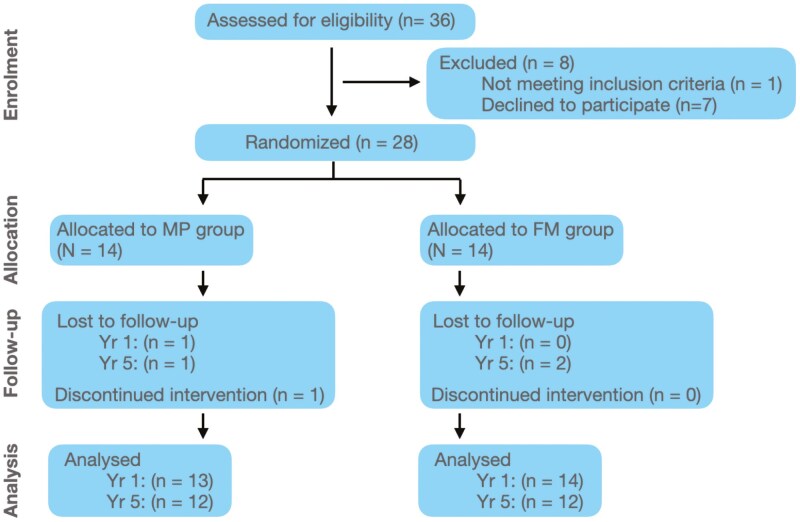
Flow diagram of patients’ allocations in the trial.

One patient, initially assigned to the MP group, was later excluded due to non-cooperation leading to discontinuation of treatment. Three additional patients, two from the FM group and one of the MP group, were lost to follow-up before the 5-year time-point (Fig. 6).

### Recruitment

Patient recruitment occurred from December 2016 to September 2018. Scans were performed in three phases: initial scans (T0) from February 2017 to September 2018, first follow-up (T1) from February 2018 to August 2019, and second follow-up (T2) from October 2022 to February 2024. (supplementary file 1 for more details)

### Baseline data

Twenty-eight patients (14 males, 14 females; mean age 9.7 ± 1.3 years) participated in the study. The FM and MP groups were age- and gender-matched (Table 1). Most patients were at CVMI stages 2 or 3, with one patient at stage 1. Initial Class III malocclusion severity was comparable between groups (Table 2; supplementary files 2 and 3 for complete data). The randomization process was adhered to rigorously, and there was no violation. The baseline comparisons are provided solely for descriptive purposes and not to infer statistical significance, in alignment with CONSORT principles.

**Table 1.** Number, age and duration of treatment.

	N (Female/Male)			Age (mean)			Duration of treatment (mean) (months)	
Group	T0	T1	T2	T0	T1	T2	T0-T1	T0-T2
FM	14 (7/7)	14 (7/7)	12 (7/5)	9 y, 6 mo	10 y, 0 mo	15 y, 5 mo	12 mo	64 mo
MP	14 (7/7)	13 (6/7)	12 (5/7)	9 y, 7 mo	10 y, 7 mo	15 y, 4 mo	11,7 mo	65 mo

FM: Facemask; MP: Mentoplate; T0: baseline; T1: 1 year follow-up; T2: 5 year follow-up.

### Numbers analyzed

Twenty-eight patients (14 FM, 14 MP) underwent low dose CT at baseline (T0). At one year (T1), 27 patients (14 FM, 13 MP) completed follow-up scans. By five years (T2), 24 patients (12 FM, 12 MP) remained for analysis (Fig. 6 and Table 1).

### Outcomes and estimation

Both inter-observer (ICC: 0.96–0.99) and intra-observer (ICC: 0.93–0.99) measurements showed high reliability with no significant differences between observers. Both treatment groups (FM and MP) exhibited similar sagittal and vertical skeletal changes, as shown in Table 3. During active treatment (T1-T0), cephalometric analysis revealed maxillary advancement (SNA; FM: + 2.48, MP: + 1.99; p = .60) and slight mandibular retraction (SNB; FM: −0.86, MP: −0.64; p = .73). Both ANB (FM: + 3.36, MP: + 2.63; p = .34) and Wits (FM: + 4.42, MP: + 2.86; p = .12) measurements showed similar changes in both groups. Post-treatment follow-up (T2-T1) demonstrated comparable relapse patterns in both groups, characterized by mandibular catch-up growth (SNB; FM: + 1.63, MP: + 1.78; p = .92) and minimal maxillary advancement (SNA; FM: + 0.18, MP: + 0.88; p = .61). Vertically, no significant jaw rotations occurred during active treatment. However, in phase two, both groups exhibited counterclockwise mandibular rotation (SN-ML; FM: −3.03, MP: −1.12; p = .36), resulting in a slight increase in the posterior-to-anterior facial height ratio (PFH/AFH; FM: + 3.58, MP: + 2.25; p = .44). During active treatment, incisors responded differently between groups: the MP group showed increased proclination of both upper and lower incisors, while the FM group showed retroclination. These differences were statistically significant (U1-SN: p = .04, U1-NL: p = .02, IMPA: p = .01), though high standard deviations suggest individual responses varied widely. The differences in incisor inclination disappeared during follow-up (T2-T0). Mean follow-up time was 12 ± 1.59 months at T1 and 64.5 ± 5.21 months at T2 (Table 1) (for complete data, supplementary file 1).

**Table 3.** Cephalometric changes in facemask and mentoplate treatment groups.

Variable	T1-T0 Mean ± SD			T2-T0 Mean ± SD			T2-T1 Mean ± SD		
	Facemask	Mentoplate	p-value	Facemask	Mentoplate	p-value	Facemask	Mentoplate	p-value
Sagittal									
SNA °	2.48 ± 2.47	1.99 ± 2.01	.60	2.65 ± 2.89	2.88 ± 4.14	.88	0.18 ± 2.60	0.88 ± 3.97	.61
SNB °	−0.86 ± 1.39	−0.64 ± 1.65	.73	0.78 ± 2.57	1.14 ± 4.04	.79	1.63 ± 2.95	1.78 ± 3.96	.92
ANB °	3.36 ± 2.19	2.63 ± 1.40	.34	1.90 ± 3.07	1.71 ± 2.65	.87	−1.46 ± 2.40	−0.93 ± 2.33	.59
Wits (mm)	4.42 ± 2.11	2.86 ± 2.59	.12	3.33 ± 2.50	1.50 ± 3.45	.15	−1.08 ± 2.43	−1.36 ± 3.92	.84
Vertical									
SN-ML °	0.52 ± 1.48	−0.46 ± 2.05	.20	−2.52 ± 4.17	−1.58 ± 5.34	.63	−3.03 ± 4.79	−1.12 ± 5.19	.36
SN-NL°	−0.18 ± 2.66	−0.71 ± 3.66	.69	−0.08 ± 2.63	−0.08 ± 5.52	1.00	0.10 ± 3.64	0.63 ± 4.55	.75
NL-ML°	0.73 ± 2.09	0.24 ± 4.48	.74	−2.42 ± 4.79	−1.83 ± 5.30	.78	−3.14 ± 4.45	−2.08 ± 4.53	.57
PFH/AFH	−0.58 ± 1.44	0.25 ± 1.76	.22	3.00 ± 4.02	2.50 ± 3.61	.75	3.58 ± 4.29	2.25 ± 3.93	.44
Dental									
U1 -SN	−3.44 ± 5.63	1.90 ± 6.26	.04*	5.74 ± 6.16	5.03 ± 11.14	.85	9.18 ± 3.92	3.13 ± 7.96	.03*
U1-FH	−2.90 ± 4.93	2.43 ± 7.55	.05	5.67 ± 6.93	6.34 ± 11.16	.86	8.57 ± 4.36	3.92 ± 7.43	.07
U1 -NL	−4.40 ± 4.86	1.27 ± 6.42	.02*	5.38 ± 7.10	4.16 ± 12.19	.77	9.78 ± 4.76	2.89 ± 8.48	.02*
IMPA	−3.13 ± 4.19	2.49 ± 6.07	.01*	3.61 ± 7.63	1.42 ± 8.31	.51	6.74 ± 7.24	−1.08 ± 9.99	.04*

T1 (1 year), T2 (5 year), FM (facemask), MP (mentoplate). Results are described in median ± standard deviations. Intergroup comparison is performed using unpaired two-sided samples t-test, *: p < .05.

(T0 was subtracted from T1; T0 was subtracted from T2 and T1 was subtracted from T2. This means that a positive value indicates more protrusion whereas a negative value indicates retrusion).

### Ancillary analyses

No ancillary analyses were done.

### Harms

In our MP patients’ group, no loosening of the plate was observed. Although some issues with the anchor hooks (fracture or mucosal irritation) were encountered, none led to treatment cessation.

## Discussion

The following study is the first RCT to compare the long-term skeletal and dental effects of FM and MP therapy in combination with HH-Alt-RAMEC. A 2D cephalometric approach was applied instead of three-dimensional analysis as vertical changes and intermaxillary changes are difficult to quantify using 3D volumetric analysis. Moreover, it allowed a more direct comparison with the existing evidence, where lack of 3D values exists in literature.

The comparison of FM and MP groups cephalometric outcomes showed no significant differences in the sagittal and vertical skeletal dimensions. These findings align with prior studies that compared HH with MP and FM at a short-term follow-up without Alt-RAMEC protocol [45]. Despite previous studies suggesting that the use of symphyseal plates could yield improved vertical control [43–45], recent research indicates that vertical control depends primarily on upper jaw anchorage [33]. Our study confirms this finding, as both groups showed effective vertical control during active treatment, despite using different lower jaw anchorage methods but identical HH devices. Effectiveness of Rapid Palatal Expansion (RPE) for maxillary protraction remains debated, though it may enhance skeletal effects by mobilizing midfacial sutures [34, 35]. The Alternate Rapid Maxillary Expansion and Constriction (Alt-RAMEC) protocol claims to achieve greater and faster skeletal changes by further stimulating upper jaw growth through circum-maxillary suture opening [33, 36, 37]. Whether skeletal anchorage through HH in the maxilla produces greater sagittal skeletal effects remains unclear due to conflicting evidence in the literature [33, 48, 49]. Adding Alt-RAMEC to the HH-device shows no additional advantages [33], which is confirmed in our short term results. Our protocol, using HH with Alt-RAMEC shows similar SNA, SNB, ANB, and wits measurements changes compared to previous studies, using both MP- RPE HH [45], FM-RPE HH and FM-Alt-RAMEC HH [9, 50]. This indicates that Alt-RAMEC does not improve HH treatment outcomes in pre-pubertal patients (CVMI 2-3), compared to RPE with HH. A possible explanation might be that, as the subjects were young and the circum-maxillary sutures were still patent, using skeletally anchored Alt-RAMEC may not have any additional benefit in correcting the sagittal relationship in comparison to skeletal anchored RPE. Our short-term outcomes of Alt-RAMEC protocol using face mask with hybrid hyrax (FM-HH) showed similar results compared to face mask with tooth-borne expander (FM-TBE) and Alt-RAMEC in prepuptertal patients [33, 50]. This suggests that in young patients (CVMI 2-3), using Alt-RAMEC with a TBE may be as effective as skeletal anchorage with hybrid hyrax.

Evidence indicates also less dento-alveolar compensation when HH is used as compared to tooth born expanders (TBE) [4, 33, 48]. In the short term (T1-T0), incisor inclination differed significantly between the MP and FM groups. The MP group showed mandibular incisor protrusion, while the FM group exhibited retrusion. This was expected and aligns with previous reports [3]. The FM group also showed unexpected maxillary incisor retrusion, while the MP group demonstrated protrusion. Though these differences were statistically significant, there was considerable variation in individual responses as depicted in the amount of SD. By the long term (T2-T0), the FM group’s incisor retrusion had reversed due to significant more relapse during phase 2 (U1-SN: p = .03; IMPA: p = .04). No long-term differences in incisor inclination remained between groups (T2). The initial differences likely stemmed from chin cup pressure during treatment, which caused lower incisor retrusion and, through occlusal interaction, upper incisor retrusion in the FM group. These differences disappeared after chin cup pressure was stopped.

Our treatment protocols showed similar or better outcomes compared to other skeletal anchorage methods, including BAMP [51, 52] and miniscrew [48, 52] protraction. The type of anchorage device appears less important [27] than the patient’s age at treatment initiation. Although BAMP protocols typically begin at an older age and include retention therapy during phase 2, our earlier intervention without retention therapy did not lead to increased relapse. In fact, our long-term results were comparable or slightly superior to long term BAMP outcomes [29, 51].

**Figure 7.**
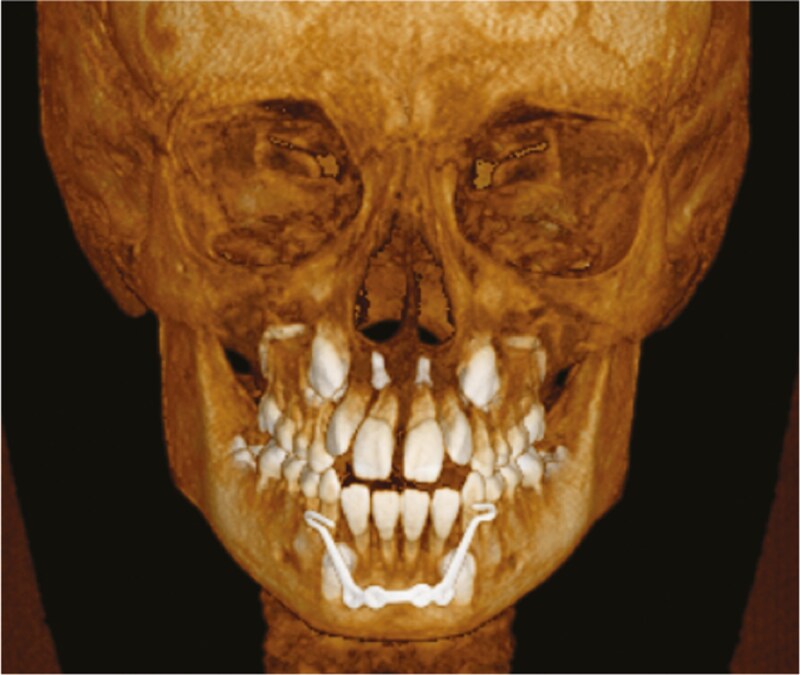
Position of the mentoplate with impacted canines in the lower jaw.

The findings of the present study indicated that MP protocol did not yield significantly superior outcomes. The MP’s effectiveness was comparable to that of FM therapy, thereby suggesting that it could serve as a feasible alternative to FM, which is currently viewed as the standard clinical treatment in growing class III patients. The MP protocol could be a suitable alternative when FM may not be the best solution due to compliance issues or anatomical limitations. Despite ongoing debates, initiating treatment at an early age appears to yield more skeletal effects [34–37]. The MP can be inserted before the eruption of the mandibular canines, facilitating an early commencement of Class III treatment and eliminating the need for FM (Fig. 7). In terms of the stability of MP as a bone anchor, this treatment option rarely experiences plate loosening, most likely due to the plate’s positioning, fixation, and form in the mandible’s basal bone. It is a single-piece construction offering simultaneous traction on both sides into the basal bone, thereby preventing plate loosening. In our MP patients’ group, no loosening of the plate was observed. Although some issues with the anchor hooks (fracture or mucosal irritation) were encountered, none led to treatment cessation. The inclusion of MP in the orthodontic toolkit is especially recommended when customizing treatment to accommodate each patient’s unique needs and preferences. Furthermore, the HH device also showed no loosening of mini-screws during treatment, as these screws are rigidly connected to the HH device. Considering the better vertical control, less dento-alveolar compensations using HH and potential negative effects of TBE-Alt-RAMEC on buccal bone thickness [53], skeletal anchorage with hybrid hyrax is the preferred choice of anchorage in the upper jaw. Alt-RAMEC does not improve the results in young patients, whether this is the case in patients when treatment is started at later age is unknown.

A key factor in both treatment options is patient compliance. It is recommended that patients use the facemask 14 hours a day and intra-oral elastics 24/7 in the first months of treatment. It has been suggested that patient compliance is easier obtained using bone-anchors and intra-oral elastics. As in all other studies, it would have been most desirable being able to objectively measure the exact wear times of the elastics for maxillary protraction.

The strength of the study was the first time prospective RCT-based reporting of the long-term comparison of HH + FM and HH + MP therapy.

## Limitations

The study had certain limitations. Firstly, a limited number of patients were included in the study. Although sample size calculation was performed, a larger sample might be required to reach a definitive conclusion owing to the unpredictable growth issues. Significant patient attrition occurred at the five-year follow-up, also in the mentoplate (MP) group. This was unexpected since MP patients typically return for hardware removal. Although all mentoplates were eventually removed, the varied timing of these procedures extended beyond the T2 timepoint. Therefore, these patients did not undergo low dose CT and were excluded from the study to avoid potential bias. Secondly, the outcome analysis was solely based on 2D cephalometry. This approach may oversimplify results by focusing on measurable outcomes while overlooking aesthetic quality and genetic factors. Class III malocclusion is complex, influenced by multiple genes, ethnic background, and environmental factors like muscle function and nutrition [54]. Individual variation in growth patterns and genetic predisposition for class III malocclusion may significantly influence treatment outcomes, regardless of the intervention chosen. Our sample size, while statistically adequate, may not fully represent the spectrum of genetic variability in Class III patients. This randomized controlled trial (RCT) focused specifically on appliance design and mechanics and its findings challenge the common assumption that bone anchors provide superior results despite being more invasive. The study results enable direct comparison with previously published data on this topic. While our 5-year follow-up provides valuable insights, the ultimate stability trough completion of growth cannot be guaranteed, as some patients may experience late mandibular growth. Future studies should focus on 3D cephalometric and volumetric analysis studies, which could serve as a roadmap for virtual 3D diagnosis and treatment planning. Finally, the impact of the treatment protocols on soft tissue was not assessed. It is also recommended to perform future studies for assessing compliance and pain perception associated with MP.

## Generalisability

Our results are primarily applicable to patients in the mixed dentition phase (average 9.7 ± 1.3 years) with moderate skeletal discrepancies. The findings may not extend to patients with severe skeletal class III malocclusions or those at different developmental stages.

As this trial was conducted in a single center with an experienced surgeon and orthodontists, results might vary in different clinical settings. We used a standardized Alt-RAMEC protocol and force system. Different expansion protocols or force magnitudes might yield different outcomes.

## Interpretation

Early class III treatment with HH + MP skeletal anchorage does not seem to induce a better outcome as compared to HH + FM protocol. The stability of the therapeutic results seems to be consistent for both treatment groups at a long-term follow-up. These findings challenge the common assumption that bone anchors necessarily provide superior results despite being more invasive. This is particularly relevant for clinical decision making, as it suggests that the choice between FM and MP should be based on individual patient factors rather than presumed mechanical advantages.

## Supplementary Material

cjaf011_suppl_Supplementary_Files_1

cjaf011_suppl_Supplementary_Files_2

cjaf011_suppl_Supplementary_Files_3

## Data Availability

The data used to support the findings of this study are available within the article, its Supplementary materials and online repository (Meyns, Joeri (2025), ‘Long-term Comparison of Maxillary Protraction with Hybrid Hyrax-Facemask vs Hybrid Hyrax-Mentoplate Protocols Using Alt-RAMEC: A 5-Year Randomized Controlled Trial’, Mendeley Data, V1, doi: 10.17632/2p6mf2fn2f.1)

## Registration

This Randomized Clinical trial was registered at www.ClinicalTrials.gov (ID: NCT02711111) Ethical approval has been granted by the Ethics Committee at the Ziekenhuis Oost Limburg, Belgium (EudraCT B371201629565) (13/12/2016).
